# Food insufficiency and Twitter emotions during a pandemic

**DOI:** 10.1002/aepp.13258

**Published:** 2022-04-03

**Authors:** Stephan J. Goetz, Connor Heaton, Muhammad Imran, Yuxuan Pan, Zheng Tian, Claudia Schmidt, Umair Qazi, Ferda Ofli, Prasenjit Mitra

**Affiliations:** ^1^ Northeast Regional Center for Rural Development Penn State University State College Pennsylvania USA; ^2^ Department of Agricultural Economics, Sociology, and Education Penn State University State College Pennsylvania USA; ^3^ College of Information Sciences and Technology Penn State University State College Pennsylvania USA; ^4^ Qatar Computing Research Institute Hamad Bin Khalifa University Doha Qatar

**Keywords:** food insecurity, machine learning, Twitter sentiments, U.S. states

## Abstract

The COVID‐19 pandemic initially caused worldwide concerns about food insecurity. Tweets analyzed in real‐time may help food assistance providers target food supplies to where they are most urgently needed. In this exploratory study, we use natural language processing to extract sentiments and emotions expressed in food security‐related tweets early in the pandemic in U.S. states. The emotion *joy* dominated in these tweets nationally, but only *anger*, *disgust*, and *fear* were also statistically correlated with contemporaneous food insufficiency rates reported in the Household Pulse Survey; more nuanced and statistically stronger correlations are detected within states, including a negative correlation with joy.

The SARS‐Cov‐19 pandemic not only raised concerns about the resilience of the global food system (Laborde et al., 2020; Goetz et al., 2020; Johansson et al., 2021; Ridley & Devadoss, 2021 for U.S. fruits and vegetables; Charlton & Castillo, 2021 for labor supply issues), but it also caused higher household food insufficiency (FI) rates (Ziliak, 2021; Jablonski et al., 2021; Ahn & Bailey Norwood, 2021; Gundersen et al., 2021; Tian et al., 2021; Zhou et al., 2021); here we use the terms food insecurity and food insufficiency interchangeably. Identifying specific sub‐national locations where problems exist either in terms of food access (demand or ability to pay) or food availability (supply issues) is important for policymakers and welfare agencies concerned with the population's food security status. In the United States, state‐level information about household FI was collected weekly during the pandemic by the U.S. Census Bureau in the Household Pulse Survey (HPS).^1^ FI is defined in the survey as *the percent of adults in households where there was either sometimes or often not enough to eat in the last 7 days*.

To address problems such as pandemic‐related food insufficiency, affordable and readily available real‐time data are needed. Surveys tend to be costly, and results are available only with delays (see also the discussion in Ahn & Bailey Norwood, 2021). As Coble (2020, p. 295) points out, “Einav and Levin (2014) emphasize that economics has moved toward greater focus on empirical work and that the data revolution occurring in our society makes available new, large‐scale data.” However, while big data analysis is increasingly common in some areas of agriculture, such as crop and soil science (Bronson & Knezevic, 2016; Coble et al., 2018; Huang et al., 2018), its adoption in the social sciences has been more gradual. Important sources of big data for researchers include grocery store scanners as well as social media such as Twitter streams, which have been used in studies designed to minimize supply chain waste (Mishra & Singh, 2018), improve efficiency (Singh et al., 2018), or to assess responses to COVID‐19 along supply chains (Sharma et al., 2020). Other studies use social listening tools such as NetBase, which collects data from discussion streams, social networking, Twitter, product reviews, and others. Widmar et al. (2020) used NetBase to capture social media posts to analyze U.S. consumers' perceptions of egg‐laying hen housing, and Jung et al. (2021) used it to analyze food safety media attention related to flour.

Twitter offers near real‐time access to public user posts, which have been shown to provide insights into user behavior, emotional state, and sentiment (Buettner, 2017). Researchers have studied public sentiment on Twitter during the COVID‐19 pandemic (Abd‐Alrazaq et al., 2020; Barkur & Vibha, 2020; Dimitrov et al., 2020; Lwin et al., 2020) primarily by applying “off‐the‐shelf” models (Gupta & Yang, 2018; Loria et al., 2014; Thelwall et al., 2010) to sets of COVID‐19‐related tweets. These endeavors mostly use general‐purpose sentiment analysis models such as SentiStrength (Thelwall et al., 2010), CrystalFeel (Gupta & Yang, 2018), or TextBlob (Loria et al., 2014). The models use manual methods such as a dictionary to map each word to a sentiment score, counts and/or frequencies of positive and negative words, and part of speech tags. While these studies provide valuable information, off‐the‐shelf models are based on *classical* machine learning (ML) approaches (e.g., support vector machines, logistic regression, and naive Bayes) that use hand‐crafted features (e.g., bigrams, trigrams, and word‐sentiment dictionaries) without fully leveraging recent advances in deep learning and natural language processing (NLP) (Deng & Liu, 2018; Goldberg, 2017; Goodfellow et al., 2016).

In this article, we analyze sentiments and emotions expressed in tweets within the United States to identify potential associations between real‐time data and the status of household food insecurity at the state‐level. We propose an improved, general‐purpose sentiment algorithm that applies state‐of‐the‐art NLP techniques to a purposefully curated set of tweets to gauge online *sentiments* (i.e., positive, negative, or neutral) and *emotions* (i.e., anger, disgust, fear, joy, sadness, surprise, or neutral) with respect to food insecurity during the COVID‐19 pandemic that are geo‐tagged using a novel approach. We suggest this as the first step in a line of work leading to a potential early‐warning system for assisting populations with food security‐related concerns.

We find that after the *neutral* sentiment, *joy* is the dominant emotion expressed in food (in‐) security related tweets in the first 6 months of the pandemic, possibly reflecting relief that the U.S. food system did not collapse despite dire early warnings (Barrett, 2020; Macias, 2020; Poppick, 2020). We then examine simple (unconditional) correlations between these real‐time data and state‐level household FI conditions as measured by the HPS over time. At the national level, only *fear*, *anger*, and *disgust* have a statistically significant and positive correlation with changes in contemporary food insufficiency in a state. As expected, the *negative* sentiment is correlated with food insufficiency rates at the state level. While these national level correlations are small (<0.200), we obtain much larger ones (>0.500) at the level of individual states.

## METHODS: AUTOMATED TWEET EXTRACTION AND SENTIMENT/EMOTION TAGGING

In this section, we briefly describe the tools used (a) to extract and geo‐tag food insecurity‐related tweets to the 50 states and Washington, DC; and (b) the ML model architecture used to attach sentiments and emotions to each tweet.

### Extraction of tweets related to food insufficiency in U.S. states

We start with the GeoCOV19 dataset (Qazi et al., 2020) that consists of hundreds of millions of multilingual tweets posted worldwide related to the COVID‐19 pandemic and extract all English‐language tweets posted between February 1, 2020 and August 31, 2020. This period includes Phase I of the Household Pulse Survey administered by the U.S. Census, from April 27, 2020 to July 20, 2020. As tweets in this dataset cover a wide range of topics related to COVID‐19 and originate from different countries, we only select tweets related to FI posted from the United States. For this purpose, we first apply a filter using the geolocation information available with tweets, including geocoordinates, place tags, and location mentions in the text (see Qazi et al., 2020 for details).

Next, we manually curated a set of (*N* = 138) key terms that are relevant to the topic of food insecurity or insufficiency. These are shown in Table S1 in the supplemental materials and include the terms food availability, shortage, food acceptability, and food adequacy. These terms were then used to form logical expressions and to retrieve tweets that match one or more of them. The filtering steps yielded 1,275,463 tweets over the period February 1, 2020–August 30, 2020, that are geolocated in the United States and related to food insecurity or insufficiency.

**FIGURE 1:**
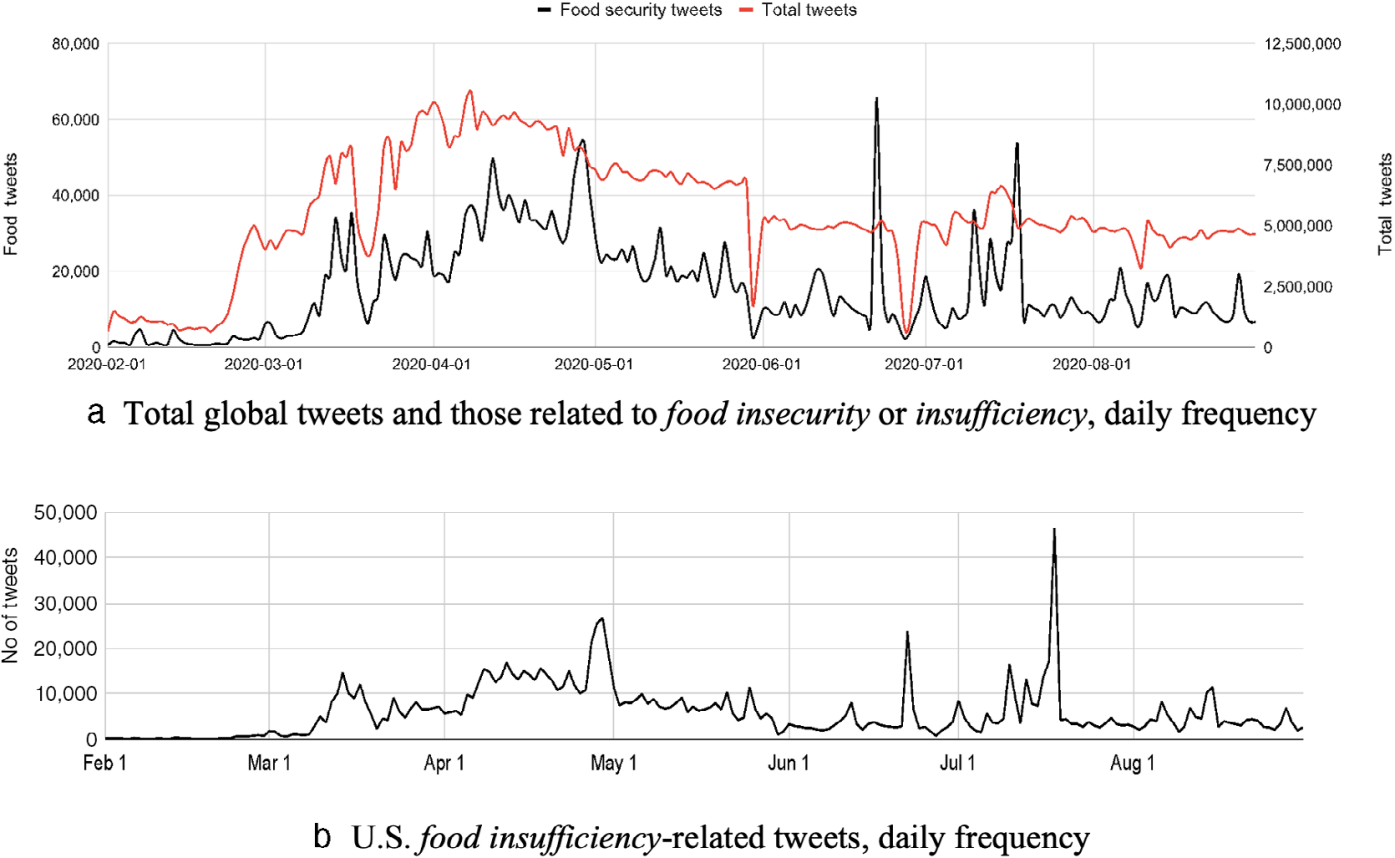
Global and U.S. food‐insufficiency‐related tweets. Food security/insecurity are based on terms in table S1 of supplemental materials *Source*: Qazi et al., 2020 and authors [Color figure can be viewed at wileyonlinelibrary.com]

Figure 1a shows the daily frequency of all tweets and food insufficiency‐related tweets world‐wide over the period shown, which starts before and ends after Phase I of the HPS. Figure 1b shows all food insufficiency related tweets specific to the United States. A gradual increase is evident in the volume of tweets starting from the last week of February 2020, similar to that observed globally, with a significant peak in the last week of April, and other peaks occurring in June and July. The U.S. peaks tend to occur slightly later than those in the global data series. We aggregate these daily tweets to a weekly basis that matches the 12 weeks of data from the Household Pulse Survey Phase I.

**FIGURE 2:**
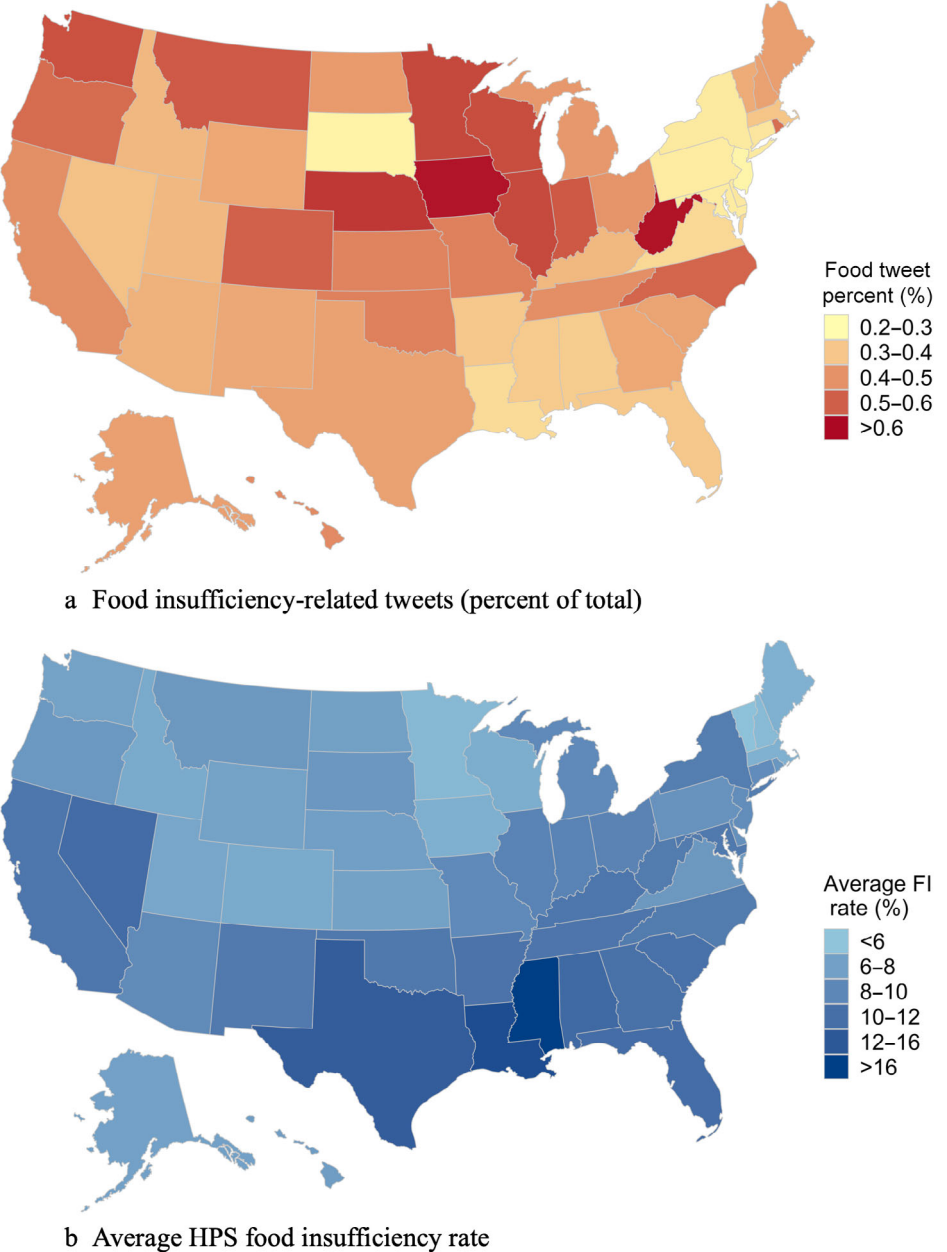
State‐level distribution of food insufficiency‐related tweets and Household Pulse Survey (HPS) food insufficiency rate. FI, food insufficiency. *Source*: Qazi et al. (2020) for (a) and household pulse survey for (b), and authors [Color figure can be viewed at wileyonlinelibrary.com]

In Figure 2a we map the distribution of food sufficiency‐related tweets by U.S. states normalized by all tweets and averaged over the 12‐weeks corresponding to those of the HPS Phase I food insufficiency data. High tweet rates in West Virginia and Iowa stand out over this period, along with low rates in South Dakota and Pennsylvania, among other states. This suggests that the importance of food insufficiency in motivating tweets in the former two states was greater during the first few months of the pandemic compared to the latter, but we do not know whether they reflect an absence of food sufficiency, or not.^2^ In particular, these numbers tell us nothing about the sentiments or the emotions associated with the tweets; these could have been positive, negative, or neutral in the case of sentiments, and emotions varying from anger and fear to relief (joy) could be motivating or captured in the tweets. To detect these nuances, we next applied classifiers that use a language model (LM) based on artificial intelligence.

### Language modeling

We use the Bidirectional Encoder Representation from Transformers (BERT) LM described in Devlin et al. (2019) to process the food insecurity‐related tweets.^3^ BERT uses the text contained in each tweet to construct an internal representation of the tweet that is then used to classify various qualities of the tweet (such as emotions or sentiments). In our case, given a food insufficiency related tweet, BERT produces as output the sentiment(s) and emotion(s) conveyed by the text of the tweet. The term “bidirectional” refers to the fact that text strings are read both forwards and backwards for context during the data processing.

We briefly describe relevant aspects of BERT here; the original paper by Devlin et al. (2019) provides a more thorough description. BERT was developed on the principle that the meaning of words is defined by their context. To “understand” or interpret language, however, BERT needs to “learn” about it, just as humans do. To accomplish this, BERT originally “learned” about the English language by being set to browse the entire internet instead of reading a dictionary (more akin to previously used approaches). Browsing the internet, BERT can discern the meaning of words by “seeing” *how they are used* instead of being “told” *what they mean*.

As part of the automated learning, BERT trains itself by creating small *fill‐in‐the‐blank* tests for itself to ensure it understands the language being conveyed^4^; this is the ML aspect of the procedure. The hypothesis is that if BERT can correctly fill‐in‐the‐blank, then it sufficiently understands the English language. For example, when BERT encounters the sentence “I love you” online, it creates the fill‐in‐the‐blank test of “I love _____” for itself. Using the broader context of the text string that has been read, the likelihood is high that this blank is correctly filled with the word “you.” If instead the fill‐in‐the‐blank test were “I go to the gym every day, I love _____” then BERT may fill the blank with a word such as “exercise.” By creating millions of such fill‐in‐the‐blank tests for itself as it browses the internet, BERT builds an “understanding” of the English language.

After many days of browsing the internet and self‐testing, BERT has developed a strong understanding of the English language, approaching human‐level performance on fill‐in‐the‐blank tests. Given this strong general understanding of the language, BERT is an excellent starting point for more fine‐grained language‐related tasks, such as those we pursue here. We use the BERT language model as the backbone of our architecture.

When given a piece of text to process, BERT first converts the text from words to tokens, or *subword pieces*, and each token has a corresponding embedding. Doing so allows BERT to understand a wider range of language with less overhead—instead of having to learn a different representation for each word in a language, BERT only needs to learn a representation for the tokens and can understand words as the union of one or more tokens. A special classification token called [CLS] is then added to every input. During processing, BERT draws upon all parts of the corresponding input to saturate the [CLS] token with key aspects of the language given as input. The processed [CLS] embedding can then be used as a condensed representation of the original input text.

Once the input representations have been obtained, they are processed by BERT. In each layer the token representations are updated based on their context—that is, the surrounding tokens in the input. The exact way in which BERT processes inputs is based on their content; subsequent representations of prepositions are informed by the corresponding object, passive auxiliary verbs by the verbs they modify, and direct objects by their verbs (Clark et al., 2019). The output of BERT is a rich, contextualized representation of the static embeddings it was initially given.

To process a piece of text, BERT includes as an output a representation of the entire text. This representation is a 1×H vector, where *H* is the “hidden size” of BERT (see Devlin et al., 2019 for details). Then, for each task that BERT is trained to perform two *heads* are added, one a *projection head* and another a *prediction head*. Here *head* refers to a matrix of learnable weights. The *projection head* is first used to project the generic text representation to a task‐specific embedding‐space of size H. Concretely, the *projection heads* are of size $H\times H_{t}^{\prime }$, where $H_{t}^{\prime }$ is the embedding space for task *t*. Then, the output of the *projection head* is passed through the *prediction head* to make the ultimate predictions for task *t*. Concretely, the *prediction heads* are of size $H_{t}^{\prime }\times G_{t}$ where *G*_*t*_ is the output size of task *t*.

### Training

We use BERT's general knowledge of language as a starting point to fine‐tune the model for two specific NLP tasks, sentiment analysis and emotion detection. In fine‐tuning the model, we train it to perform two other tasks as well—stance detection (Gorrell et al., 2018) and information disclosure (Jaidka et al., 2020). Much like a developing athlete can benefit from playing multiple sports—using proficiencies developed in one sport to improve ability in another—so does our model (Zhang & Yang, 2021). We briefly describe the datasets used for training in sentiment analysis and emotion detection below.

Each task used to train our model is a classification task, so our model minimizes a criterion function, which is the average cross entropy for all tasks during training. The cross entropy is calculated as described in Equation (1), where *B* is a batch of training records, {*T*} denotes the set of training tasks present in the batch, *B*(*t*) is the subset of batch records corresponding to task *t*, *y* is the set of ground‐truth labels, and *p* the model predictions. The inner summand calculates the per‐task entropy while the outer summand averages the loss across tasks. Denote |*T*| to be the number of tasks in the set {*T*}. Then the cross‐entropy function is as follows:

(1) $$H\left(y,p\right)=-\sum_{t\in \left\{T\right\}}\frac{1}{\left|T\right|}\sum_{i\in B\left(t\right)}y_{t,i}\log\left(p_{t,i}\right)$$

### Sentiment detection

We used three datasets to train our model to perform sentiment analysis: (1) Stanford Sentiment Treebank (Socher et al., 2013), (2) Sentiment 140 (Go et al., 2009), and (3) SemEval 2017 Task4A (Rosenthal et al., 2017). The Stanford Sentiment Treebank is a collection of 215,154 phrases from online movie reviews, each annotated for sentiment by three authors. We use the 5‐class version of the dataset (SST‐5) where the sentiment of each tweet is given on a five‐point Likert scale.

The Sentiment140 and SemEval2017 Task4A datasets are like SST‐5 in many regards but were originally sourced from Twitter. The dataset for SemEval2017 Task 4A contains around 50,000 tweets that have been annotated by crowd workers as expressing either negative, neutral, or positive sentiment. Sentiment140 is the largest sentiment analysis dataset used in this study, consisting of 1.4 M tweets. Tweets in Sentiment140 are given noisy binary annotations derived from the presence of emoticons in the original tweet. For example, a tweet containing “:)” is labeled as positive while a tweet containing “:(” is labeled negative. We removed emoticons from the tweets prior to inclusion in the dataset so as not to bias our model. Were the emoticons not removed, the model would not be required to learn about the language—if it detected a “:)” it could simply give the positive label without looking at the surrounding language. Example records from each dataset and their corresponding sentiment label are presented in Table S3.

### Emotion detection

For this study, we used the GoEmotion dataset, a collection of 58,000 Reddit comments annotated as expressing one or more of 27 possible emotions or neutrality by three annotators (Demszky et al., 2020). As our study is not intended to develop a fine‐grained emotional understanding, we use the mapping provided by these authors to map the 27 original emotion classes to six emotions, plus neutral, which encompass the 27 original classes. These six universal emotions are often referred to as Ekman emotions (Ekman, 1992), and datasets for emotion detection typically adopt a coding scheme derived from these emotions. All humans can feel these six emotions.

After the mapping, records in this dataset contain one or more of seven possible labels that our model learns to detect: (1) anger, (2) disgust, (3) fear, (4) joy, (5) sadness, (6) surprise, or (7) neutral. Example records and their corresponding emotion labels are presented in Table S4.

### Food insufficiency‐related examples

To illustrate the output of the above analysis for our application using BERT, Table 1 shows sample tweets that are related to some aspect of food insecurity classified as expressing a negative, neutral, or positive sentiment, along with one or more of seven emotions, including a neutral emotion. Specific probabilities are attached to each tweet for the different sentiments and emotions. For example, the first tweet is classified as negative with a probability 98.4%, with very small odds that it is neutral (1.5%) or positive (0.2%). Likewise, the chances are very high (81.6%) that the main emotion expressed in this tweet is anger, with disgust and the neutral emotion having much smaller odds. Neutral is a leftover bin that is used as a residual to arrive at 100% classification both for sentiments and emotion.

**TABLE 1:** Sample food insufficiency‐related tweets and corresponding sentiments and emotions

Tweet no.	Tweet text	Sentiments (%)			Competing emotions (%)						
		Negative	Neutral	Positive	Anger	Disgust	Fear	Joy	Sadness	Surprise	Neutral
1	@CAgovernor STOP SHUTTING DOWN OUR FOOD SUPPLY, YOU COWARDLY TYRANT	98.4	1.5	0.2	81.6	6.4	1.3	1.7	2.7	0.7	5.7
2	Why dont stores sell the darn masks by the door, I cant run all over town looking for a mask just to get a carton of milk and dog food. https://t.co/f97e2lJI68	96.1	3.5	0.4	64.5	4.7	1.6	3.1	4.5	2.5	19.1
3	The lockdown crowd is going to destroy the country. I'm not kidding. They're misleading you into thinking the virus is a single variable problem. IT ISN'T. Hospitals are going bankrupt, ppl are avoiding doctors, the food supply is strained, & we are on the cusp of mass poverty	97.4	2.2	0.4	56.4	7.7	3.8	3.9	10.9	3.1	14.2
4	Oddly enough, the grocery store is out of Nestle Quik. Damned virus. #COVID #fun #COVID19 #coronavirus https://t.co/xYG9IS3o2m	93.6	2.7	3.7	32.1	16.6	10.3	9.4	13.5	4.8	13.4
5	Went to the grocery store yesterday for the first time in weeks and it was terrifying. I wore a mask, gloves, wiped down my cart but saw many customers wearing no protective gear, touching everything. I cant imagine how the workers must feel at these stores.	67.5	9.6	23.0	2.4	3.4	28.5	8.8	36.8	14.2	5.9
6	I love this. Big Love to all the heroes of the food supply chain. Thanks for sharing some of their stories @nstomatoes https://t.co/XkBQN114eQ	1.1	2.8	96.0	0.0	0.0	0.0	99.9	0.0	0.0	0.0
7	God bless Americas food supply chain, from the producers to the distributors to the grocers: The food supply chain, they say, remains intact and has been ramping up to meet the unprecedented stockpiling brought on by the coronavirus pandemic	8.8	18.9	72.3	0.2	0.0	0.1	91.6	0.2	0.5	7.4
8	Things Ive never done on a wednesday afternoon. Make butter from organic milk. Perks of #lockdown I can say. This is from Buffalo. Native cow tomorrow (process is too tedious) https://t.co/1TqYyAxQn5	14.0	16.2	69.8	1.4	0.3	0.4	82.9	0.7	1.3	13.0
9	@RonnieLouise2 Yep…next week. And I'm donating some to our local food pantry too!	2.3	15.1	82.6	0.4	0.1	0.1	63.9	0.2	1.5	33.8
10	My fav Asian grocery store is now closed because of #Coronavirus …its so sad. Now I cant make spring rolls anymore	97.3	2.5	0.2	10.2	2.6	2.4	2.0	73.8	2.5	6.6
11	I have to close commissions since I have too much on my queue. But im still struggling. And im still trying to afford groceries and food for pets. Car repairs and a new monitor. Everything helps…Im working day and night already, and I need help… https://t.co/LlZmhppkX9	87.0	9.6	3.4	2.3	0.8	2.5	12.6	74.0	1.9	5.9
12	omg the only grocery store I feel comfortable shopping at rn lost power and all the refrigerated food went bad and I just want CHEEZ	93.0	6.6	0.4	11.3	1.9	4.1	5.5	51.5	11.8	13.9
13	Why is everyone making banana bread during this pandemic? I'm not complaining, but why is this such a consistent pattern for everyone rn? should I be making banana bread? Am I missing something?	71.8	24.7	3.4	0.7	0.1	0.4	0.6	0.6	96.4	1.2
14	ok…so…voting is more dangerous than going to the grocery store? or going to an abortion clinic?	73.8	24.8	1.4	5.8	0.3	0.7	3.1	1.3	79.1	9.7
15	Where is all the #rubbingalcohol for the public in #NewYorkCity? Been shopping around for 6 weeks and nothing. Shouldn\u2019t we have it by now & before we hit the streets? Weren\u2019t distilleries helping on that front?\u1f64F@NYGovCuomo @NYCMayor @Walgreens @cvspharmacy @Walmart #nyc @costco	79.9	19.2	0.9	4.2	0.3	1.1	2.9	1.8	76.6	13.1
16	Food bank line in Queens https://t.co/5PHGUbcnvM	10.2	74.1	15.7	0.2	0.0	0.0	0.6	0.0	0.4	98.7
17	The 40 City‐supported meal sites will be closed Monday and open Tuesday, 10 a.m.\u2013noon. Many food sites will change schedules. Call 311 to find a nearby food pantry, or text your zip code to 800–548‐6479 for a list. More info in this press release: https://t.co/p9qGxv90V7	14.2	75.1	10.7	0.3	0.0	0.0	1.9	0.1	0.7	96.9

*Note*: The shaded areas represent the highest probability that each tweet is classified to one of three sentiments and one or more of seven competing emotions (including neutrality).

*Source*: Authors.

### Testing the model

As a reference to how well our model performs the sentiment analysis, two human annotators annotated the same 100 tweets for the sentiment. The annotators were asked to label each tweet as either *negative*, *neutral*, or *positive*. In the three‐class setting, the annotators had an agreement of 0.41 in terms of Cohen's kappa, which measures the overall agreement between two annotators classifying items into a given set of categories (Kvålseth, 1989). For tweets that neither annotator labeled as *neutral*, the annotators had an agreement of 0.9 in terms of Cohen's kappa. We compare the performance of our model with that of two common “off‐the‐shelf” models on food‐related tweets where both annotators agreed on the annotation.^5^ Comparing our results with those of common *off‐the‐shelf* models and human annotators, we find that our model outperforms these common models and achieves a high level of agreement with the human annotators.

For most tweets, BERT calculates a relatively high probability that a particular emotion dominates. Competing emotions appear with relatively high odds only for tweets No. 4 and 5 in Table 1, such as anger, disgust, and sadness for tweet No. 4, and fear and sadness for tweet No. 5. Tweet No. 6 illustrates how the emotion *joy* can be associated even with a food insecurity‐related tweet. As can be seen below, *joy* is in fact the most strongly expressed among the seven emotions. Tweet No. 6 is classified with almost complete certainty in the *joy* category, and it suggests gratitude for the fact that the U.S. food supply chain continued to function despite dire early warnings and predictions of collapse. Tweets No. 7–9 similarly show why the emotion of *joy* is plausible even in the face of a potentially catastrophic pandemic.

## DATA DESCRIPTION AND ANALYSIS

### FI from the HPS

**FIGURE 3:**
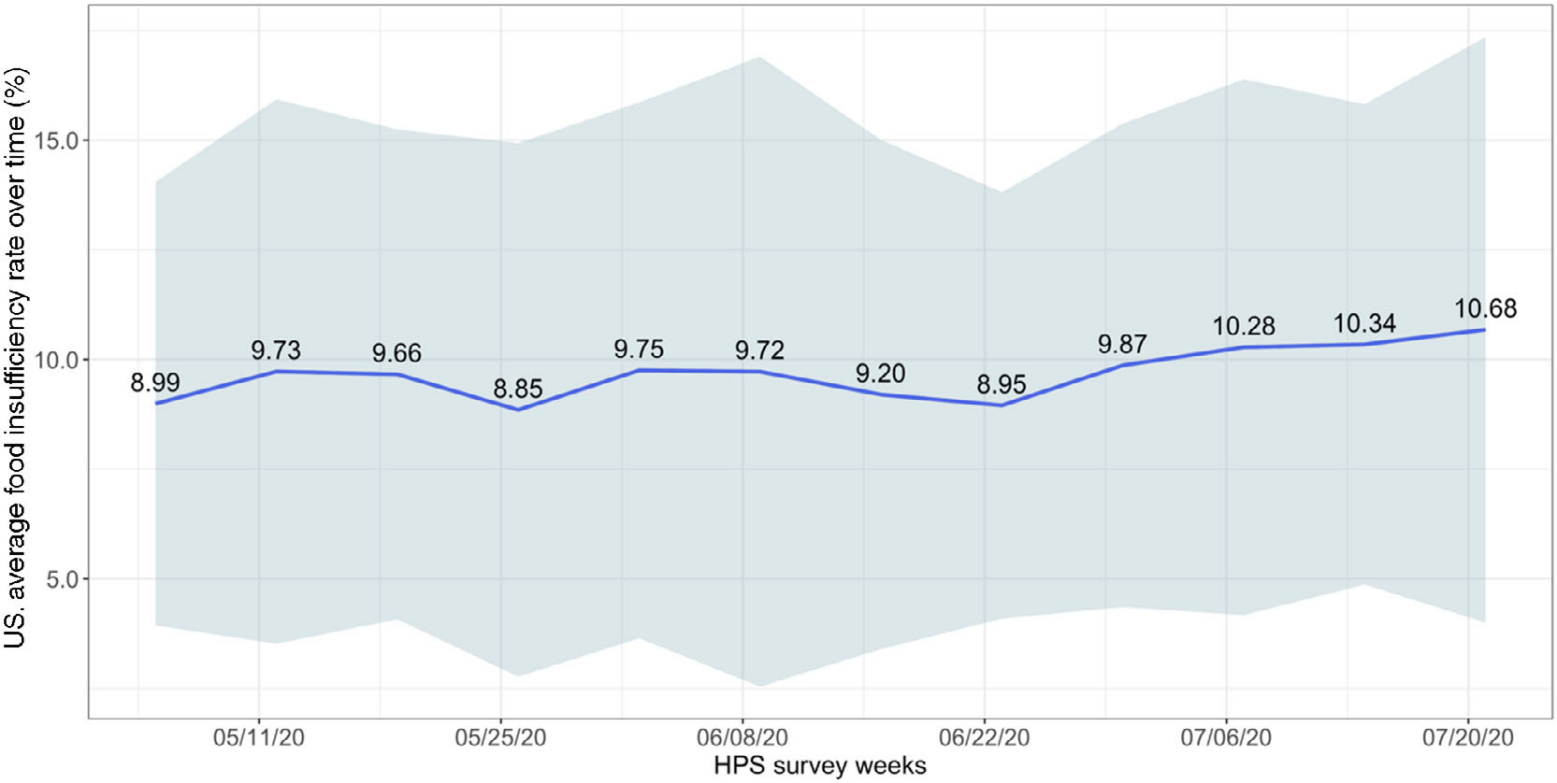
Average (and variation across states in) food insufficiency, the United States. *Source*: Household Pulse Survey (HPS) and authors' calculation. The blue line represents the averaged values of food insufficiency rates across states, and the shaded area represents the confidence interval with two standard deviations from the mean [Color figure can be viewed at wileyonlinelibrary.com]

Figure 3 shows the average and variation across states of changes in the state‐level household FI rates over time, based on Phase I HPS data, covering the end of April through July 2020. The average household FI rate at the end of Phase I was higher (10.7% of respondents indicating FI) than at the beginning (8.99%), which is consistent with the worsening effects of the pandemic over time, even as household stimulus payments started to roll out soon after April 15.

Figure 2b maps the distribution of the FI variable at the state level averaged over the roughly 3 months of data collected in Phase I of the Household Pulse Survey. The highest FI rate was recorded in Mississippi, which also leads the U.S. states in terms of poverty rates. High FI rates are also recorded in other southern states, especially Louisiana and Texas, as well as Nevada, along with New York and California. Low rates were recorded in Minnesota, Iowa, Massachusetts, and the northern New England states.

Figure S1 in the supplemental materials shows tweet‐derived sentiments and emotions across each of the states, averaged over the 12‐week period. The emotion *anger* was prominent in tweets emanating from numerous southern states as well as Idaho and Wyoming. Anger rates were comparatively low in Iowa as well as New York and the southern New England states. In contrast, *fear* was expressed most strongly on average over this period in Iowa, perhaps ironically given the state's status as being in the nation's breadbasket. *Joy, surprise*, and the *negative* sentiment were also expressed most commonly in that state, which along with Nebraska and West Virginia also had the highest share of FI‐related tweets. California, known for its general state of happiness,^6^ had the lowest score on the *joy* emotion. New Jersey, South Dakota, and Pennsylvania had the lowest shares of tweets dealing with food insecurity (Figure 2a). The emotions of *disgust* and *sadness* were strongest in Oklahoma, while the adjacent state of Arkansas had one of the lowest *sadness* scores. Figure S2 shows line graphs for the top and bottom three states in terms of emotions and the percent of tweets related to FI, to provide a sense of the relative variation in these variables over time.

### Sentiments and emotions from Twitter

**FIGURE 4:**
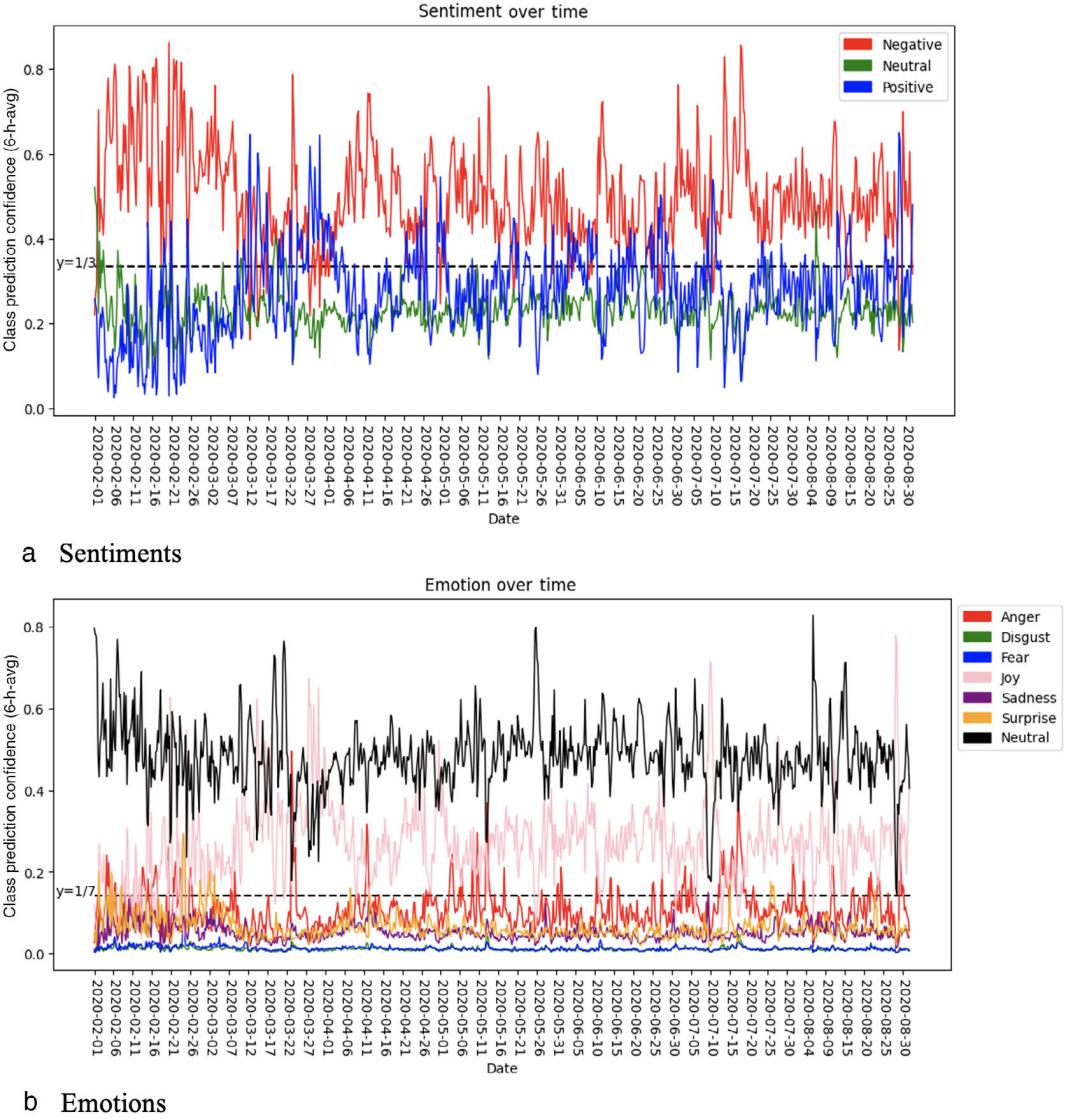
Food‐related Twitter data averaged in 6‐h buckets, the United States. Panel (a) shows the predicted shares of tweets classified into three sentiments, and panel (b) shows the predicted shares of tweets classified into seven emotions. *Source*: Authors [Color figure can be viewed at wileyonlinelibrary.com]

The *negative* sentiment dominated the FI‐related tweets, especially at the beginning and near the end of Phase I. The *positive* sentiment shows an inverted pattern to the dominant sentiment, moderated by the *neutral* sentiment. The sentiments expressed in these tweets, averaged in 6‐h windows, are presented in Figure 4a. In Figure 4b, we plot the Twitter emotions data over time, averaged across the states. The strongest emotion (or lack thereof) recorded is that of *neutral*, followed by *joy*, which also spikes sharply toward the end of the period shown, and *anger*. The prominence of the emotion *joy* is on the surface surprising, but may as already noted reflect relief over the fact that the nation's food supply was not affected as adversely as initially predicted. Descriptive statistics for each of these variables are presented in Table S5.

### Correlation analysis results

National level simple pairwise correlation coefficients among the variables of interest are shown in Table 2. Although the sizes of the coefficients are relatively small, the emotions of *fear*, *anger* and *disgust* are positively correlated with contemporaneous household FI, with *disgust* and *anger* each showing a value of about 0.16 that is significant at below the 1% level. This is not surprising given that these two emotions, in the context of food insecurity‐related tweets, are virtually indistinguishable from one another (the simple correlation is 0.945). *Disgust*, *anger*, and *fear* are also strongly correlated. In addition, the *negative* sentiment is positively correlated with FI (statistically significant but weakly, at 0.106).

**TABLE 2:** Pairwise correlations of food insufficiency (FI) rate and tweets emotions and sentiments, the United States

Variables	FI rate	Fear	Anger	Disgust	Joy	Sadness	Surprise	Negative	Neutral	Positive
FI rate	1.000									
Fear	0.104**	1.000								
Anger	0.160***	0.749***	1.000							
Disgust	0.162***	0.873***	0.945***	1.000						
Joy	−0.050	−0.515***	−0.441***	−0.469***	1.000					
Sadness	0.064	0.636***	0.376***	0.505***	−0.213***	1.000				
Surprise	−0.028	0.380***	0.134***	0.184***	−0.230***	0.294***	1.000			
Negative	0.106***	0.710***	0.787***	0.763***	−0.672***	0.442***	0.165***	1.000		
Neutral	−0.099**	−0.622***	−0.703***	−0.710***	−0.049	−0.486***	−0.156***	−0.493***	1.000	
Positive	−0.071*	−0.492***	−0.538***	−0.507***	0.797***	−0.255***	−0.108***	−0.892***	0.047	1.000

*** *p* < 0.01,

** *p* < 0.05,

* *p* < 0.1.

*Source*: Authors.

The fact that *joy* is the most common emotion and yet not statistically correlated with FI over time nationally suggests that it did not change on a weekly basis in the same way as the household FI situation. Instead, we argue that this reflects a shared feeling of gratitude or relief that food supplies generally remained steady early in the pandemic, even if specific foods were not available, such as particular cuts of meat, types of bread or ice cream flavors.

Some of the other national‐level correlation coefficients in Table 2 also are revealing, although generally as expected. For example, the *negative* sentiment is strongly correlated (>0.700) with *fear*, *anger*, and *disgust*. It is also negatively correlated (−0.672) with joy. Table 2 also shows that the neutral sentiment is strongly negatively correlated with *fear*, *anger*, and *disgust*, suggesting that these strong feelings are unlikely to coincide with a sentiment of *neutrality*, as might be expected. At the same time, even though the correlation coefficients in Table 2 are statistically different from zero, they tend to be small, suggesting relatively low correlations.

To further explore these relationships, we calculated the coefficients of correlation between emotions and FI on a state‐by‐state basis (Table 3). Focusing first on the emotion of *fear*—which we expect to be associated with a lack of food for reasons either of access (e.g., lack of income) or availability (e.g., supply bottlenecks)—we obtain statistically significant high (>0.500) and positive correlation coefficients for California (0.626), Illinois (0.577), New York (0.866), Texas (0.670) and Wisconsin (0.641). In 12 of the 15 cases where the correlation coefficient is of moderate size (i.e., between 0.30 and 0.49), it is also positive, indicating that FI in a state is associated with a higher level of *fear*, as expected.

**TABLE 3:** State‐level correlations between food insufficiency (FI) rates, FI‐related tweets, and emotions

State	%FI	Anger	Disgust	Fear	Joy	Sadness	Surprise
Alabama	−0.691**	0.210	0.166	0.173	−0.062	0.230	−0.099
Alaska	−0.082	0.014	−0.001	−0.031	0.183	−0.080	−0.382
Arizona	−0.011	0.676**	0.685**	0.402	0.100	0.277	−0.202
Arkansas	0.023	0.161	0.366	0.484	0.392	0.293	0.014
California	−0.263	0.700**	0.705**	0.626**	−0.714***	0.533*	−0.314
Colorado	−0.242	−0.081	−0.132	−0.314	0.086	−0.020	−0.130
Connecticut	−0.374	−0.315	−0.338	−0.227	−0.074	−0.334	0.342
Delaware	−0.110	0.202	0.226	0.168	0.230	0.347	−0.113
District of Col.	−0.002	−0.013	0.102	0.150	0.087	0.196	0.199
Florida	−0.163	−0.008	−0.075	−0.102	−0.159	−0.020	0.156
Georgia	0.078	0.415	0.354	0.384	−0.271	0.449	0.488*
Hawaii	−0.429	0.403	0.452	0.362	−0.108	0.425	0.244
Idaho	0.352	0.438	0.480	0.260	0.223	0.219	0.227
Illinois	−0.051	0.511*	0.566*	0.577**	−0.428	0.325	0.522*
Indiana	−0.233	−0.299	−0.345	−0.465	0.270	−0.388	−0.310
Iowa	−0.382	0.149	0.129	0.056	−0.327	−0.205	0.109
Kansas	−0.133	0.151	0.345	0.352	−0.525*	−0.025	0.178
Kentucky	−0.485*	−0.172	−0.230	−0.034	0.007	0.015	0.127
Louisiana	0.145	0.374	0.260	0.011	0.217	0.047	0.017
Maine	0.172	−0.271	−0.224	−0.076	0.428	0.095	−0.219
Maryland	0.293	0.025	−0.028	0.024	−0.061	0.240	0.497*
Massachusetts	0.638**	0.110	0.134	−0.075	0.230	0.344	0.151
Michigan	−0.110	−0.024	−0.042	0.121	−0.094	−0.139	0.148
Minnesota	−0.469	0.500*	0.459	0.427	−0.040	0.364	0.578**
Mississippi	−0.135	0.124	0.194	0.232	−0.255	0.382	0.104
Missouri	−0.420	−0.010	−0.039	−0.010	0.138	0.414	0.188
Montana	−0.651**	0.238	0.057	−0.278	0.453	−0.467	−0.300
Nebraska	0.159	0.104	0.058	0.198	0.355	0.510*	0.005
Nevada	−0.326	0.354	0.395	0.280	−0.119	0.177	−0.420
New Hampshire	−0.211	−0.415	−0.341	−0.067	0.008	0.277	−0.043
New Jersey	0.069	−0.259	−0.210	−0.165	0.002	−0.337	0.388
New Mexico	0.191	0.443	0.531*	0.426	−0.509*	0.459	−0.010
New York	0.436	0.752***	0.845***	0.866***	−0.600**	0.297	0.271
North Carolina	0.032	0.054	0.066	−0.069	−0.041	−0.135	−0.247
North Dakota	−0.697**	−0.053	−0.256	−0.288	0.347	−0.370	0.013
Ohio	−0.200	−0.151	−0.145	−0.079	0.286	−0.016	0.160
Oklahoma	−0.176	0.108	0.309	0.273	−0.392	0.481	−0.254
Oregon	0.003	0.256	−0.100	−0.332	0.153	−0.179	−0.612**
Pennsylvania	0.410	0.613**	0.533*	0.375	−0.073	−0.141	−0.180
Rhode Island	0.669**	0.490*	0.544*	0.404	0.148	0.439	−0.131
South Carolina	0.623**	−0.018	−0.013	−0.030	0.364	0.313	−0.120
South Dakota	−0.302	0.342	0.260	0.383	−0.156	−0.074	0.241
Tennessee	−0.451	0.098	0.164	0.200	0.076	0.148	−0.041
Texas	−0.119	0.662**	0.712***	0.670**	−0.043	0.612**	−0.148
Utah	0.511*	−0.046	−0.092	−0.252	0.059	−0.017	−0.431
Vermont	−0.260	0.28	0.490*	0.471	0.088	0.543*	−0.427
Virginia	−0.014	0.427	0.435	0.467	−0.007	0.350	0.501*
Washington	−0.024	−0.081	0.148	0.101	0.075	0.032	0.186
West Virginia	0.074	−0.195	−0.197	−0.261	−0.058	−0.398	−0.139
Wisconsin	−0.109	0.592**	0.575*	0.641**	−0.047	0.537*	0.092
Wyoming	−0.245	0.567*	0.343	0.124	−0.213	−0.234	0.109

*Note*: Green shading is for coefficients that are statistically different from zero; dark gray is for coefficients ranging in 0.300–0.490.

*** *p* < 0.01,

** *p* < 0.05,

* *p* < 0.1.

*Source*: Authors.

In 12 states, we also see statistically significant, positive correlation coefficients between FI and the emotions of *anger* or *disgust*. There are again 15 states in which the coefficient is of moderate size, although not significant, and here again, the sign of the coefficient is negative in only three states. For the emotions *joy* (and *sadness*) we find that in the four (five) states where the coefficient is statistically different from zero, it is as expected negative (positive). Table 3 also shows that the share of tweets related to food insufficiency can have strong negative or positive correlations with actual food insufficiency rates within states.

Thus, while further analysis is needed, our initial results suggest that there is potential in using real‐time tweets to begin to assess in which states food insufficiency may be a concern. Furthermore, while most of the correlation coefficients that are statistically significant are from states with larger cities and populations (such as California), we obtained statistically significant correlation coefficients for certain emotions even in states with smaller populations, such as Kansas (*joy*), Nebraska (*sadness*), Oregon (*surprise*) and Rhode Island (*anger* and *disgust*). In some cases, it may be desirable to use a regional approach to prediction, by combining data from states that form natural regions, such as North and South Dakota or northern New England. Other extensions may include using more refined or shorter time periods, such as biweekly or monthly data. For example, our preliminary analysis suggests that the positive correlations between FI and *anger*, *disgust* and *fear* at the national level were strongest in week 12 (July 16–21) of the pandemic, and weakest in week 8 (June 18–23). On a monthly basis, there as a positive and significant correlation between FI and *anger* or *fear* in May and July, but not in June. Last, it is also possible to identify specific locations at the substate level using longitude and latitude tweet tags; these locations could be aggregated to county or zip code levels.

## SUMMARY AND CONCLUSION

This article illustrates the application of large‐scale, real‐time data to understanding a population's sentiments and emotions relative to food insufficiency status in the early days of a pandemic. The underlying goal of the paper is to begin to shed light on whether and how such real time data could be used by policymakers and other entities to detect when and where localized food security problems may arise. Our analysis and results suggest on a preliminary basis that social media platforms such as Twitter can provide insights into the emotions and sentiments of users in a given community (state) over time‐related to a concern such as food insufficiency or security. One additional important reason for conducting this kind of analysis is to assess whether Twitter data could be used in place of a more expensive survey, such as the U.S. Census Bureau's Household Pulse Survey to predict where food insecurity may be a problem. Fully answering this question will require more robust regression analysis with an appropriate formal structural model and more control variables, allowing predictions to be made. Of course, the population using Twitter is only a subset of the U.S. population.

While we were not able to test this explicitly, because there were no severe localized disruptions to the food supply in the COVID‐19 pandemic, or regions of the country with pronounced food supply or access problems, studying tweets in the future to detect such emergencies may prove fruitful by providing an early warning system for planners, supply chain managers and policymakers; this was beyond the scope of our study. We can, however, conclude that tweets expressing *fear*, *anger* and *disgust* were individually associated with higher household food insufficiency rates. The fact that the emotion of *joy* was most frequently expressed in the food insufficiency‐related tweets over time (after the negative emotion) suggests that the Twitter population was at least relieved that food sufficiency or security issues were not more strongly felt than perhaps initially predicted by media commentators. That in turn also suggests that the U.S. food system was robust and resilient in the face of the pandemic threat.

Finally, we also suggest that this kind of analysis could potentially offer new ways of measuring well‐being in real time, including utility as expressed in joy and happiness. Consumer utility is a key concept in economics, and yet its measurement remains elusive. Sentiment and emotion analysis potentially offer economists new tools to objectively and in real‐time gauge consumer utility and thus contribute to improved policy analysis and policymaking.

## Endnotes

^1^
The HPS website is https://www.census.gov/programs-surveys/household-pulse-survey.html; as of this writing, it has been administered in three Phases; we focus on Phase I, covering the period April 27 to July 20, 2020. With the exception of the first survey wave (a 2‐week period), Phase I was administered weekly.

^2^
For example, as a “breadbasket” state, tweets originating in Iowa may have anticipated positive impacts from the pandemic due to greater global food demand, resulting in positive sentiments and emotions, whereas tweets in West Virginia, a state with high poverty rates, may have been motivated by fear or anger, and negative sentiments, given the longer history of food insufficiency in that state.

^3^
See Figure S4 in supplemental materials for a diagram of the BERT process.

^4^
“Fill‐in‐the‐blank” is one of two pre‐training tasks, the other being next‐sentence‐prediction.

^5^
See Table S2 for the results of the agreement analysis using Cohen's Kappa and Figure S3 for related Confusion Matrices.

^6^
See, for example, “Is California One of the Happiest States in the Country?” Available at https://patch.com/california/lajolla/california-one-happiest-states-country.
